# Loss of AMPK activity induces organelle dysfunction and oxidative stress during oocyte aging

**DOI:** 10.1186/s13062-024-00471-4

**Published:** 2024-04-23

**Authors:** Lin-Lin Hu, Mei-Hua Liao, Ya-Xi Liu, Chun-Hua Xing, Lan-Lan Nong, Feng-Lian Yang, Shao-Chen Sun

**Affiliations:** 1 Key Laboratory of Research on Clinical Molecular Diagnosis for High Incidence Diseases in Western Guangxi, Reproductive Medicine, Guangxi Medical and Health Key Discipline Construction Project, Affiliated Hospital of Youjiang Medical University for Nationalities, Baise, China; 2 College of Animal Science and Technology, Nanjing Agricultural University, 210095 Nanjing, China; 3 Industrial College of Biomedicine and Health Industry, Youjiang Medical University for Nationalities, 533000 Baise, Guangxi China

**Keywords:** Oocyte, Meiosis, AMPK, Mitochondria, Oxidative stress

## Abstract

**Background:**

Oocyte quality is critical for the mammalian reproduction due to its necessity on fertilization and early development. During aging, the declined oocytes showing with organelle dysfunction and oxidative stress lead to infertility. AMP-activated protein kinase (AMPK) is a serine/threonine protein kinase which is important for energy homeostasis for metabolism. Little is known about the potential relationship between AMPK with oocyte aging.

**Results:**

In present study we reported that AMPK was related with low quality of oocytes under post ovulatory aging and the potential mechanism. We showed the altered AMPK level during aging and inhibition of AMPK activity induced mouse oocyte maturation defect. Further analysis indicated that similar with its upstream regulator PKD1, AMPK could reduce ROS level to avoid oxidative stress in oocytes, and this might be due to its regulation on mitochondria function, since loss of AMPK activity induced abnormal distribution, reduced ATP production and mtDNA copy number of mitochondria. Besides, we also found that the ER and Golgi apparatus distribution was aberrant after AMPK inhibition, and enhanced lysosome function was also observed.

**Conclusions:**

Taken together, these data indicated that AMPK is important for the organelle function to reduce oxidative stress during oocyte meiotic maturation.

## Background

Oocytes are the female gametes and the oocyte maturation quality is critical for successful fertilization and following early embryo developmental competence. Organelles such as mitochondria, Golgi apparatus that perform specific functions are all necessary for mammalian oocyte meiotic maturation and fertilization. Mitochondria are responsible for generating ATP production through cellular respiration. Mitochondria also play a crucial role in the production and removal of reactive oxygen species (ROS), which is generated as a byproduct of ATP production. Aberrant ROS level will lead to oxidative stress, which refers to an imbalance between the production and removal of reactive oxygen species (ROS) in cells [27]. Oocytes contain a large number of mitochondria, which provide the energy required for oocyte spindle assembly, actin dynamics and other processes during meiosis [8]. However, the high metabolic activity and mitochondrial DNA replication during oocyte maturation make them vulnerable to oxidative stress. On the other sides, oxidative stress in oocytes can lead to mitochondrial dysfunction, impaired energy production, compromised oocyte quality, which associated with decreased fertilization rates, impaired embryo development and increased risk of chromosomal abnormalities [21]. Several conditions such as aging, environmental pollute exposure or certain diseases could induced the excessive ROS level in oocytes, resulting in oxidative damage to cellular components, including mitochondrial DNA, proteins and lipids (Y. Wang, Xing, Zhang, Pan, & Sun [32]; Xu et al [36]. To counteract the detrimental effects of oxidative stress, oocytes have various antioxidant defense mechanisms including the presence of enzymes such as superoxide dismutase (SOD), catalase, and glutathione peroxidase, which neutralize and remove ROS. Additionally, oocytes rely on antioxidant molecules like glutathione, vitamin C, and melatonin to scavenge and neutralize excessive ROS [15, 23]. Understanding the relationship between oxidative stress and mitochondrial function in oocytes is crucial for reproductive health and for developing strategies to improve oocyte quality, fertility, and assisted reproductive technologies.

Besides mitochondria, other organelles are also important to ensure oocyte maturation quality. In oocytes, endoplasmic reticulum (ER) produces proteins and lipids necessary for cellular functions and involves in the synthesis of proteins for early embryo development (Kang, Wang, & Yan [13]); Golgi apparatus modifies newly synthesized proteins and packages them into vesicles for transportation to specific cellular locations in oocytes; while lysosomes are involved in the degradation of non-functional organelles and the recycling of cellular components. Dysfunction of these organelles by external environmental exposure such as acrylamide, nivalenol could lead to oocyte maturation defects, further causing the failure of fertilization and embryo development (Y. Wang, Pan, Xing, Zhang, & Sun [30]; Wu et al [34].

AMP-activated protein kinase (AMPK) is a serine/threonine protein kinase that acts as a key regulator of cellular energy homeostasis, which is activated in response to a decrease in cellular energy levels (X. Wang, Tan, Zhang, Wu, & Shi [29]). AMPK acts as a metabolic sensor, enabling cells to adapt and survive under conditions of energy stress, and it is a heterotrimeric enzyme complex consisting of three subunits: the catalytic α subunit, and regulatory β and γ subunits. The α subunit contains the kinase domain and is responsible for substrate phosphorylation; the β subunit is involved in the stability and localization of the complex, while the γ subunit functions as an AMP and ADP binding domain. AMP binding to the γ subunit induces a conformational change that exposes the catalytic site on the α subunit, allowing it to phosphorylate downstream targets. Additionally, AMP binding promotes the phosphorylation of a specific threonine residue on the α subunit by upstream kinases such as LKB1 or CaMKKβ [9]. Activated AMPK phosphorylates numerous downstream targets, coordinating a wide range of metabolic responses to restore energy balance, including the inhibition of anabolic processes such as protein and lipid synthesis, and activation of catabolic pathways such as glycolysis, fatty acid oxidation, and autophagy [19]. AMPK also plays a role in regulating cellular processes involved in glucose and lipid metabolism, insulin sensitivity, mitochondrial biogenesis, and cell growth and survival. Dysfunction of AMPK signaling has been implicated in various metabolic disorders, including obesity, type 2 diabetes, and cancer, making it an important therapeutic target for these conditions [6]. In oocytes, AMPK is also shown to associate with aging-related oxidative stress [24, 28].

Although the roles of AMPK were revealed in multiple models, there are few studies on how AMPK affects mouse oocyte meiotic maturation, and the relationship between AMPK and oocyte aging is still largely unclear. In this study, we investigated the effects of AMPK activity loss on oocyte polar body extrusion, ROS level maintenance, mitochondria and other organelle functions. Our results showed that AMPK decreased in post ovulatory aging oocytes and AMPK activity was essential for the control of mitochondria-related oxidative stress during mouse oocyte maturation.

## Methods

### Antibodies and chemicals

All chemicals and agents used in this study were purchased from Sigma-Aldrich and Merck (St. Louis, MO) unless otherwise indicated. Anti-rabbit AMPK antibody was purchased from Abcam (ab32047, Cambridge, UK); Alexa Fluor 488 goat anti-rabbit antibody was purchased from Invitrogen (Carlsbad, CA, USA). Hoechst 33,342 (B2261) were purchased from Sigma-Aldrich and Merck (St. Louis, MO, USA). Rabbit anti-β-actin antibody (3700) were purchased from Cell Signaling Technology (Devers, MA, USA).

### Oocyte maturation and post-ovulatory aging

This study was approved by the Animal Care and Use Committee of Nanjing Agricultural University and were performed in accordance with related guidelines. 5–6 weeks old ICR mice were used to collect the ovaries, and then the oocytes were acquired at fully grown stage. The oocytes then were washed in the M2 medium for several times and were cultured in M16 medium under paraffin oil with 37 ℃ and 5% CO_2_. For post-ovulatory aging treatment, after 12-hour culture of ICR mouse oocytes, we retained the oocytes in the culture medium for additional 12 h, the time point which is well accepted in previous studies.

For COM C treatment, we first prepared COM C to dissolve in DMSO with 10 mM, and then we diluted it in M16 culture medium to the different final concentration as 5 µM, 10 µM etc., ensuring that the DMSO final concentration in the medium was no more than 0.1%.

### Fluorescence staining and confocal microscopy

For AMPK antibody staining, we performed the protocols following previous studies [12]. We first fixed the mouse oocytes with 4% paraformaldehyde for 30 min at room temperature; and then we moved the oocytes to the medium with 0.5% Triton X-100 for 20 min for permeabilization. With the blocking buffer incubation for 1 h under 1% BSA-supplemented phosphate-buffered saline (PBS), the oocytes were stained with AMPK antibody for 4 h at room temperature. Oocytes were then washed with PBS for 3 times, stained with the secondary antibody for 1 h at room temperature. With another washing, the oocytes were then stained with Hoechst 33,342 for 10 min at room temperature.

For ROS, mitochondria, ER, Golgi apparatus and lysosome detection, the mouse live oocytes were incubated in M16 medium with ROS (S0033, Beyotime), Mito-Tracker Red CMXRos (1:200) (M7512, Thermo Fisher), ER-Tracker Red (1:200) (C1041, Beyotime), Golgi-Tracker Red (1:200) (C1043, Beyotime), and Lyso-Tracker Red (C1046, Beyotime), following the operation instructions by the relative manufacture. Then the oocytes were stained with Hoechst 33,342 for 10 min at room temperature. and examined with a confocal laser-scanning microscope (Zeiss LSM900, Germany).

The glass slides were spread with the oocytes and put under the laser scanning confocal microscope (Zeiss LSM900, Germany) to scanning. All the experiments were repeated at least three times with at least 30 oocytes.

### Western blotting

Western blotting protocol was adopted based on previous descriptions [40]. About 150–200 oocytes were collected for western blotting detection. The oocytes were lysed in Laemmli sample buffer (SDS sample buffer with 2-mercaptoethanol), and then were heated at 100 ℃ for 10 min. SDS-polyacrylamide gel electrophoresis (PAGE) was performed for these samples. With the treatment of electrophoretic separation, the general proteins were moved to the PVDF membrane (polyvinylidene fluoride) (Millipore, Billerica, MA), and then the PVDF membranes were blocked with 5% non-fat dry milk in TBST (Tris-buffered saline with 0.1% (w/w) Tween 20) at room temperature for 2 h. After the washing with TBST, the PVDF membrane was incubated with anti-AMPK antibody (1:1000) antibody (1:1000), anti- actin antibody (1:4000), and anti-GAPDH antibody (1:2000) at 4 ℃ for at least 8 h. With another washing in TBST, PVDF membranes were incubated with relative secondary antibodies (1:2000) at room temperature for 1 h. Finally, the PVDF membranes were exposed to an enhanced chemiluminescence reagent (EMD Millipore, Billerica, MA, USA) and imaged by Tanon-3900 (Tanon, Shanghai, China).

### ROS, ATP content and mtDNA detection

After culture, the 30 mouse oocytes were transferred to M16 medium containing 10 pM DCFH-DA (S0033, Beyotime) and treated for 30 min at 37 °C in 5% CO_2_ incubator, and then the oocytes were mounted on petri dish and examined by confocal laser-scanning microscope (Zeiss LSM 900, Germany).

ATP assay kit (S0026, Beyotime) was adopted to measure the general ATP content in mouse oocytes, following the operation instructions by the relative manufacture. 30 oocytes were diluted with ATP releasing reagent with pure water before use. The released sample was then mixed with ATP assay mix for ATP hydrolyzing, following the bioluminescence value measurement.

To detect mtDNA copies number. 50 oocytes were used to extract the DNA with DNA extracted kit (R0083, Beyotime). Real-time quantitative PCR was performed to measure the relative content of mtDNA copy number. The primers were following our previous study: mtDNA primers: F, 5′- CCA ATA CGC CCT TTA ACA AC -3′; R, 5′- GCT AGT GTG AGT GAT AGG GTAG − 3′. β-actin primers: F, 5′- TGT GAC GTT GAC ATC CGT AA -3′; R, 5′- GCT AGG AGC CAG AGC AGT AA-3′;

### Fluorescence intensity and statistical analysis

To calculate the fluorescence intensity, we mounted the oocyte samples on the same glass slide with same setting for scanning parameters to avoid the technique errors. Image J software (NIH, Bethesda, MD) was used to measure the fluorescence intensity. Detailed methods could be found in our previous studies [22]. Specifically, due to the fact that organelles were aggregated at spindle periphery at MI stage, we measure this area to more concisely reflect the organelle siganls.

At least three independent biological repeats were performed and at least 30 oocytes were examined for each experiment. The data were presented as means ± SEMs. Statistical analysis was done by GraphPad Prism 5.0 software (San Diego, CA). Statistical comparisons were run by independent student t-tests. P value less than 0.05 was as significant.

## Results

### Expression and localization of AMPK in mouse oocytes

**Fig. 1:**
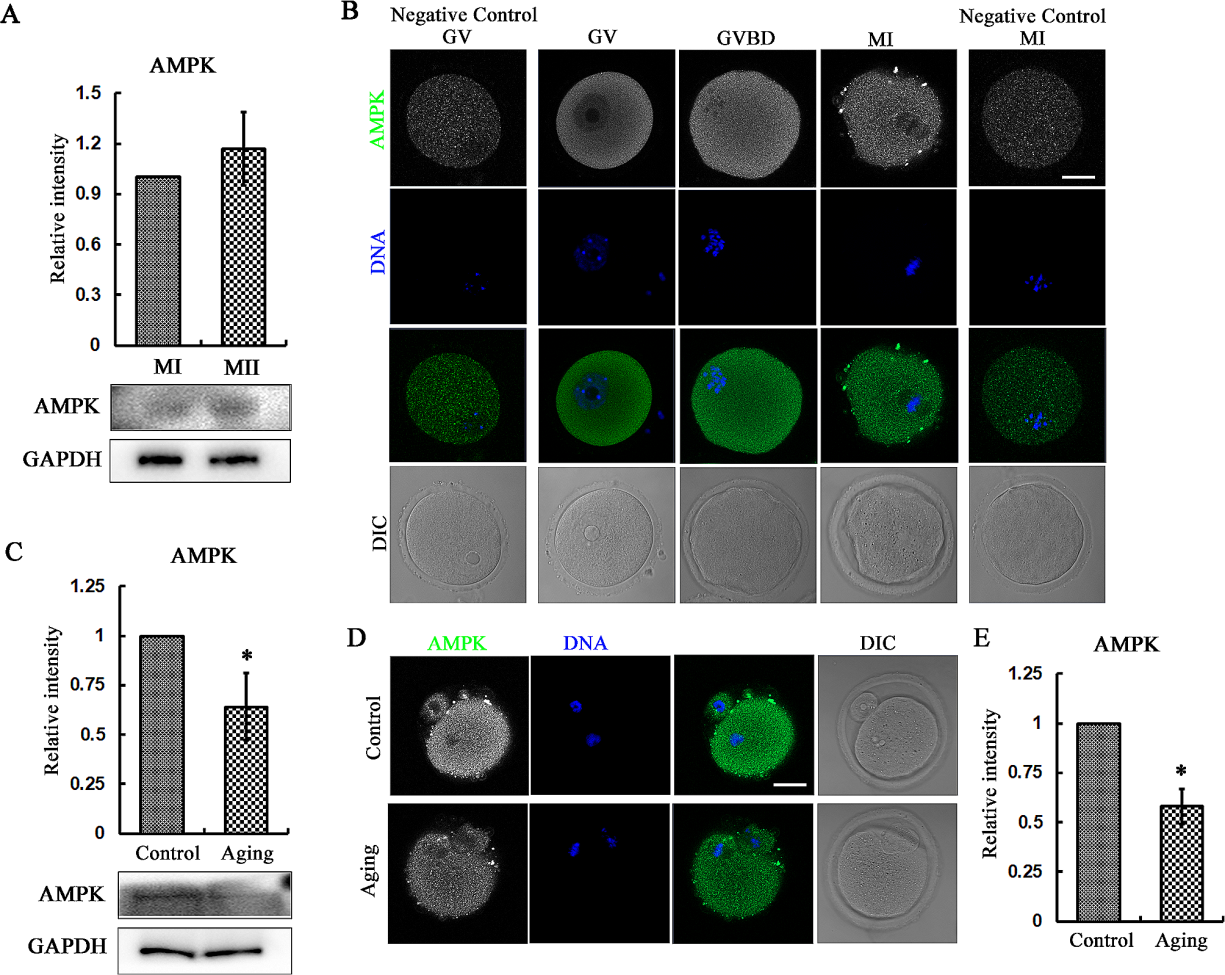
Expression and localization of AMPK in mouse oocytes. (**A**) The AMPK protein expression in metaphase I and metaphase II of mouse oocytes by western blotting. 200 oocytes were used for each sample. (**B**) The localization of AMPK during mouse oocyte maturation. AMPK localized in the cytoplasm of mouse oocytes from GV and MI stage, while there was no AMPK signal in the negative control groups. Green, AMPK; blue, DNA. Bar = 20 μm. (**C**) The AMPK protein expression in metaphase II of mouse oocytes during aging. 150 oocytes were used for each sample. **P < 0.05. (**D**) The localization of AMPK during mouse oocyte aging. In the aged oocytes, there was no difference for the AMPK localization, however, the fluorescence signals were much weaker than the fresh oocytes. Green, AMPK; blue, DNA. Bar = 20 μm. (**E**) The rate of relative intensity of AMPK. **P < 0.05

We first examined the AMPK expression in oocytes. As shown in Fig. 1A, we found that AMPK both stably expressed in metaphase I and metaphase II during meiosis in mouse oocytes (1 vs. 1.17 ± 0.22). Immunofluorescence staining data revealed that during oocyte meiosis I, AMPK uniformly localized in the cytoplasm from GV stage (Fig. 1B). While we found that during post ovulatory aging, AMPK expression decreased at the metaphase II stage (1 vs. 0.64 ± 0.17, *p* < 0.0511111) (Fig. 1C). Consistently, the fluorescence staining data also confirmed this, showing with weaker signals in the cytoplasm (Fig. 1D). This finding was also verified by the fluorescence intensity analysis data (1 vs. 0.58 ± 0.09, *p* < 0.05) (Fig. 1E). These data suggested that AMPK existed in the oocytes and showed perturbated level during oocyte aging.

### AMPK activity is essential for mouse oocyte maturation quality

**Fig. 2:**
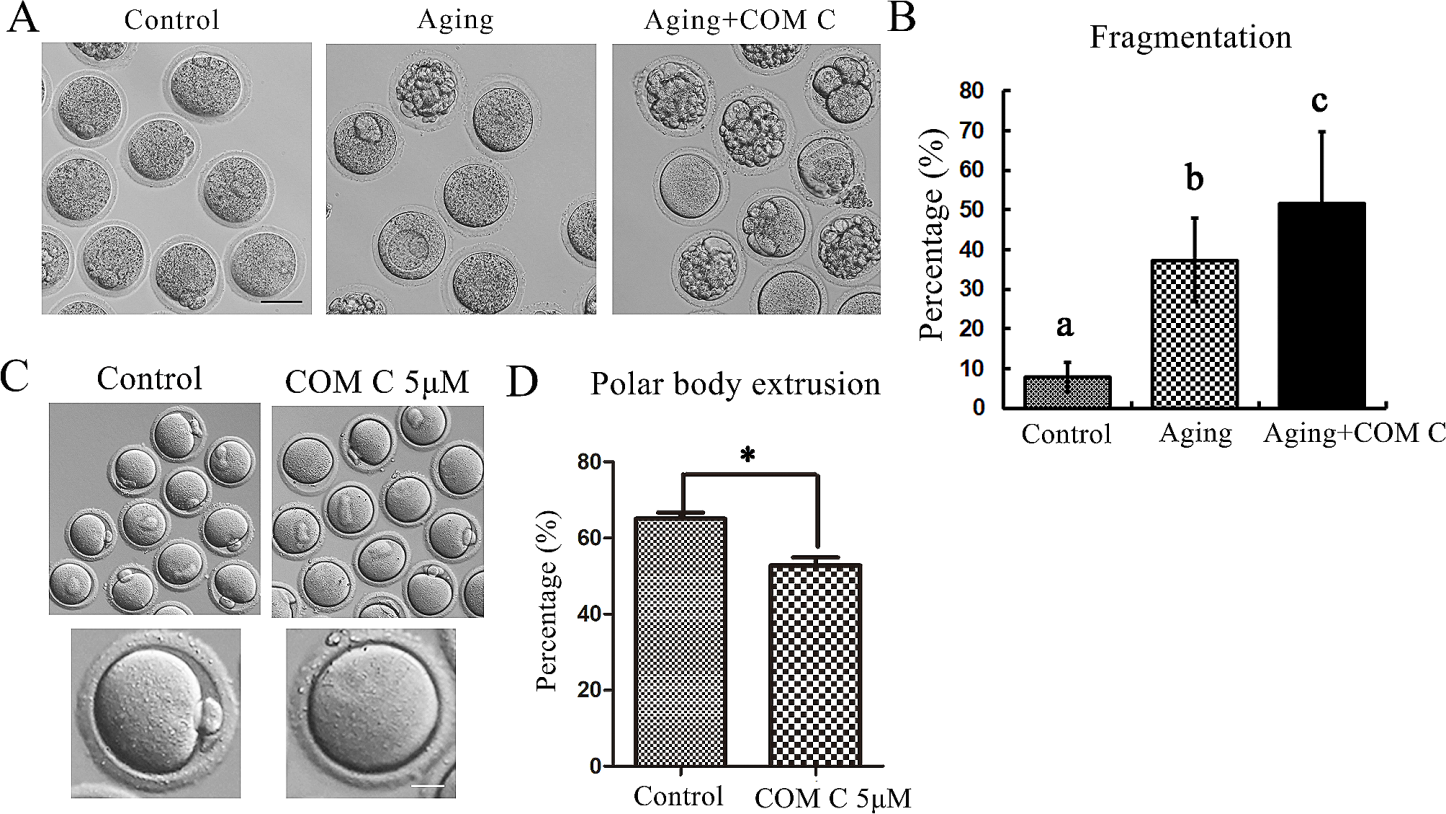
AMPK activity is essential for mouse oocyte maturation quality. (**A**) The representative images of MII oocyte morphology in the control, aging, aging + COM C groups. The oocytes showed normal polar body in the control group; however, there were fragment oocytes after maturation in the aging oocytes; while more proportion of oocytes in the aging oocytes treated with AMPK inhibitor COM C. Bar = 50 μm. (**B**) The percentage of fragmentation of oocytes in the control, aging, aging + COM C groups. *p < 0.05. (**C**) The representative images of oocytes in the control and COM C groups. Bar = 20 μm. (**D**) The percentage of polar body extrusion in the oocytes of the control and COM C groups. *p < 0.05

The decreased AMPK expression in aged oocytes might be the response to the declined metabolism since previous studies indicated that mitochondria functions were weakened in mammalian aged oocytes. To examine whether AMPK was essential for oocyte maturation, we inhibited AMPK activity by COM C treatment. Our data showed that after 12 h culture, the fresh oocytes showed normal morphology, however, in aged oocytes there was big proportion of fragmentation, while supplement with COM C in aged oocytes showed much high ratio of fragmentation (Fig. 2A). The statistical analysis data confirmed our finding (7.87 ± 3.67% for control oocytes; 37.4 ± 10.7% for aging oocytes; 51.8 ± 17.8% for aging + COM C oocytes) (Fig. 2B). We then examined the development competence of oocytes after AMPK inhibition, and as shown in Fig. 2C, loss of AMPK activity caused the failure of first polar body extrusion. The rate of polar body extrusion was significantly lower than the control group (65.00 ± 1.73% vs. 52.73 ± 2.16%, *p* < 0.05) (Fig. 2D). These data suggested that AMPK was essential for oocyte maturation.

### AMPK suppresses oxidative stress in mouse oocytes

**Fig. 3:**
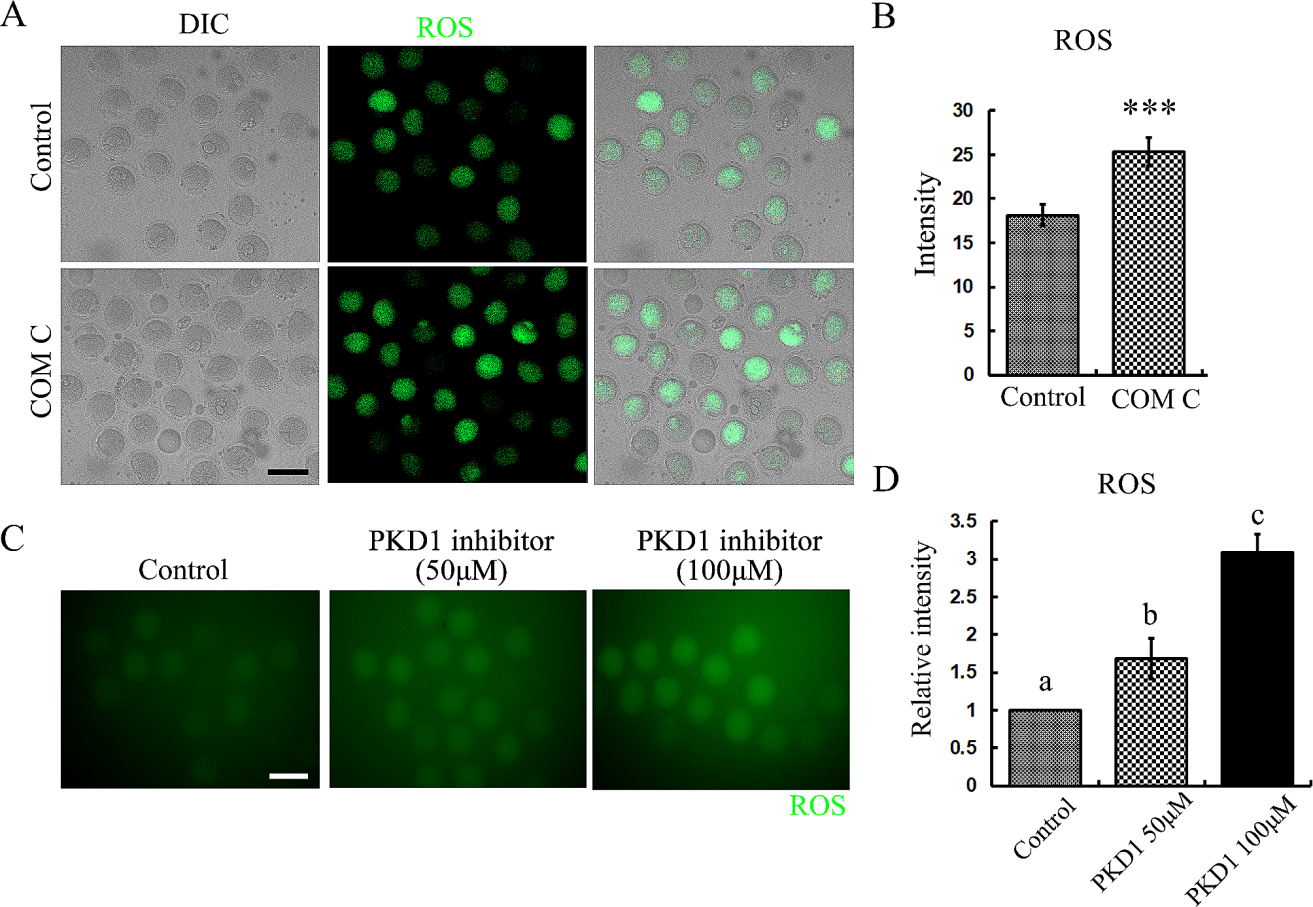
AMPK suppresses oxidative stress in mouse oocytes. (**A**) The representative images of ROS level in the oocytes of the control and COM C groups. Green, ROS. Bar = 100 μm. (**B**) The relative fluorescence intensity of ROS in the control and COM C groups. ***P < 0.001. (**C**) The representative images of ROS level in the oocytes of the control and PKD1 inhibition groups. Green, ROS. Bar = 100 μm. (**D**) The relative fluorescence intensity of ROS in the control and PKD1 inhibition groups. *p < 0.05, **P < 0.01

To explore the possible roles of AMPK in oocytes, we first examined ROS level, since several studies indicates that AMPK was related with oxidative stress in other models. As shown in Fig. 3A, there were few oocytes showed significant signals of ROS in the control group after 12 h culture; however, a big proportion of oocytes showed strong ROS signals. We also performed fluorescence intensity to qualify this, and the data was consistent with our fluorescence staining finding (25.30 ± 1.6 vs. 18.15 ± 1.2, *p* < 0.001) (Fig. 3B). To further evaluate this, we also inhibited the activity of PKD1, a molecule which was shown to be the upstream regulator for AMPK, and the results showed that increasing PKD1 inhibitor doses induced more ROS in mouse oocytes (Fig. 3C), which also was confirmed by the fluorescence intensity qualification data (1 vs. 1.68 ± 0.27 vs. 3.09 ± 0.24, *p* < 0.05) (Fig. 3D). These data suggested that AMPK was essential for ROS level maintenance during oocyte maturation.

### AMPK regulates mitochondria functions in mouse oocytes

**Fig. 4:**
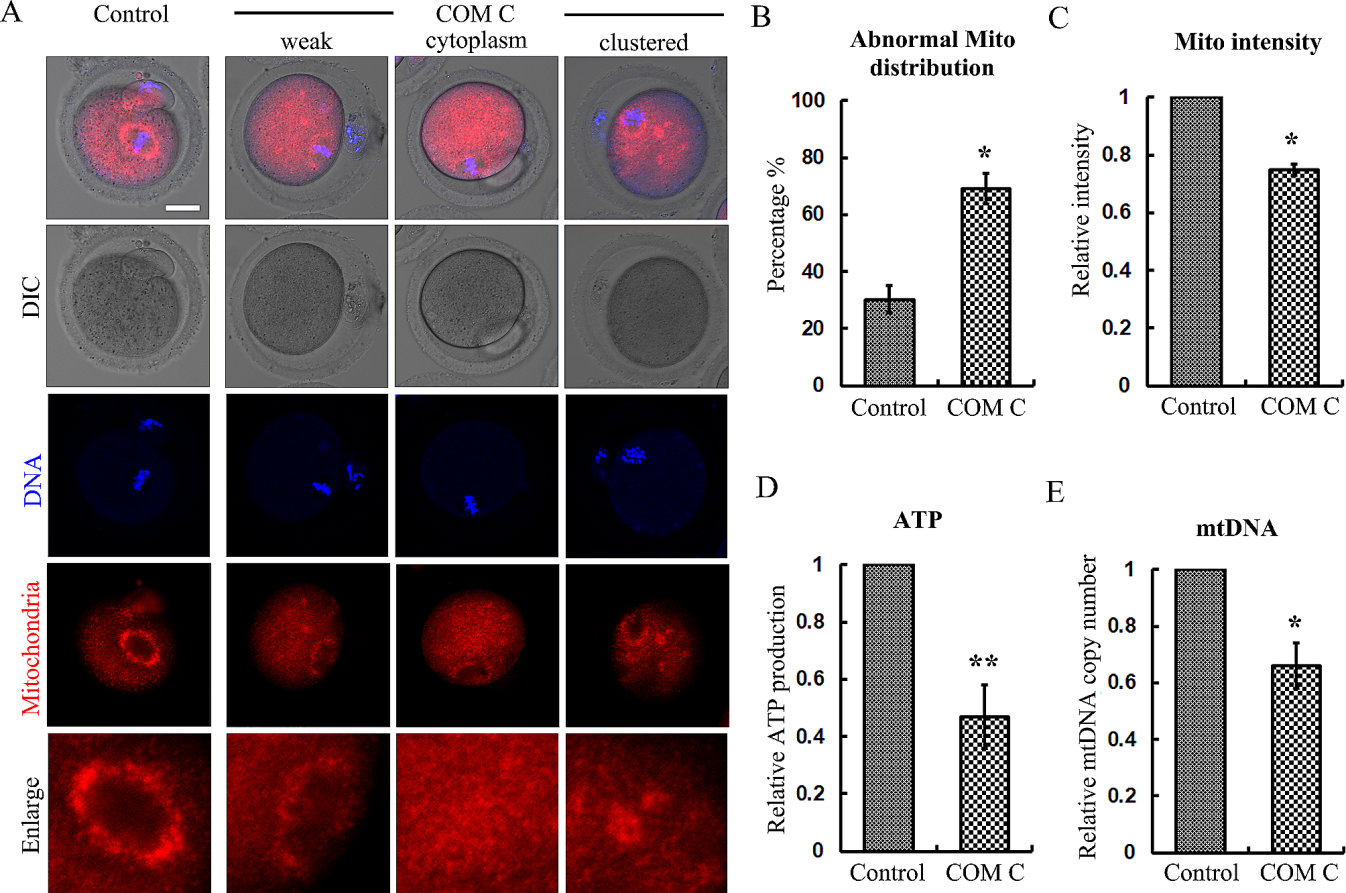
AMPK regulates mitochondria functions in mouse oocytes. (**A**) The representative images of mitochondria distribution in the oocytes after AMPK inhibition. Red, mitochondria; blue, DNA. Bar = 20 μm. (**B**) The rate of abnormal mitochondria distribution in the oocytes after AMPK inhibition. **p < 0.01. (**C**) The relative intensity of mitochondria in the oocytes after AMPK inhibition. *p < 0.05. (**D**) The relative ATP production ratio in the oocytes after AMPK inhibition. **p < 0.01. (**E**) The relative mtDNA copy number in the oocytes after AMPK inhibition. *p < 0.05

The effects of AMPK on the prevention of oxidative stress may be due to mitochondria dynamics since it is well accepted that mitochondria dysfunction was one main cause for ROS level alteration. We first examined the mitochondria distribution, and the fluorescence probe staining showed that different with the spindle periphery localization of mitochondria in the control oocytes, AMPK inhibition caused several aberrant distributions of mitochondria, such as weakened distribution at the spindle periphery, uniformly distribution in the cytoplasm, or the clustered accumulation in the cytoplasm (Fig. 4A). The rate of abnormal mitochondria distribution in the treatment group was significantly higher than the control group (30.2 ± 4.86% vs. 69.2 ± 5.26%, *p* < 0.05) (Fig. 4B). We also measured the fluorescence intensity and the statistical analysis data also confirmed this (1 vs. 0.72 ± 0.02, *p* < 0.05) (Fig. 4C). To further evaluate the roles of mitochondria, we also measured ATP production and mtDNA copy number, and the data showed that the relative ATP reproduction was significantly lower than the control group (1 vs. 0.47 ± 0.11, *p* < 0.05) (Fig. 4D); similar finding was also found for the relative mtDNA copy number (1 vs. 0.66 ± 0.08, *p* < 0.05) (Fig. 4E). These data suggested that AMPK was essential for mitochondria distribution and function during oocyte maturation.

### AMPK maintains ER and Golgi apparatus in mouse oocytes

**Fig. 5:**
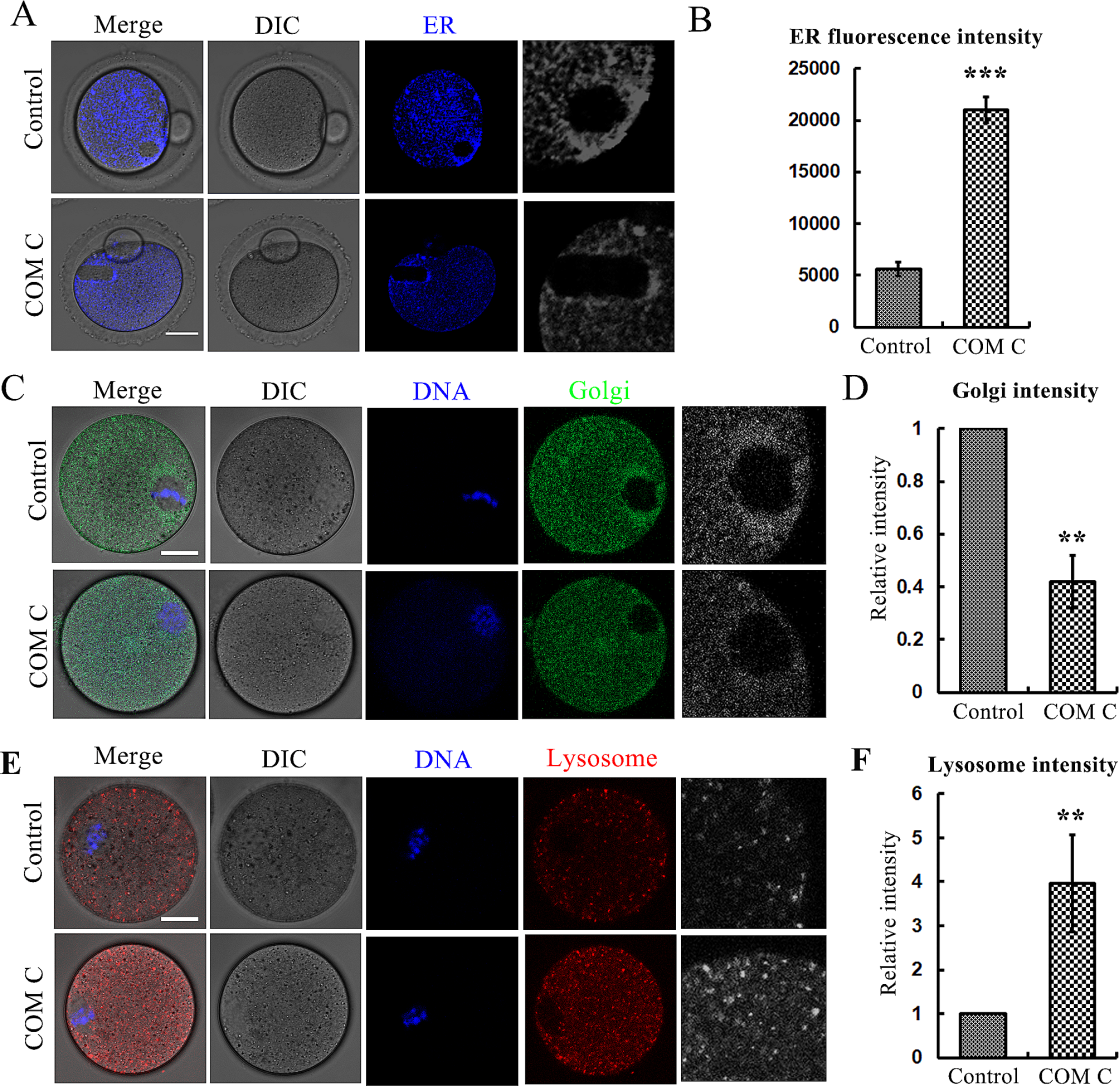
AMPK maintains ER and Golgi apparatus in mouse oocytes. (**A**) The representative images of ER distribution in the oocytes after AMPK inhibition. Blue, ER. Bar = 20 μm. (**B**) The relative ER fluorescence intensity in the oocytes after AMPK inhibition. ***p < 0.001. (**C**) The representative images of Golgi apparatus distribution in the oocytes after AMPK inhibition. Green, Golgi apparatus; blue, DNA. Bar = 20 μm. (**D**) The relative Golgi apparatus fluorescence intensity in the oocytes after AMPK inhibition. ***p < 0.001. (**E**) The representative images of lysosome distribution in the oocytes after AMPK inhibition. Red, lysosome; blue, DNA. Bar = 20 μm. (**F**) The relative lysosome fluorescence intensity in the oocytes after AMPK inhibition. **p < 0.01

Due to the effects of AMPK on mitochondria, we also examined other organelles. Our data showed that ER distribution at the spindle periphery of MII oocytes was also disrupted after the loss of AMPK activity (Fig. 5A), which was verified by the fluorescence intensity analysis (56171 ± 635.9 vs. 21,015 ± 1227, *p* < 0.001) (Fig. 5B). Similarly, the Golgi apparatus was failed to accumulate to the spindle periphery area after AMPK inhibition (Fig. 5C), and this was also confirmed by the Golgi fluorescence intensity analysis (1 vs. 0.42 ± 0.10, *p* < 0.01) (Fig. 5D). Mitochondria dysfunction and oxidative stress could lead to the autophagy, we then examined the lysosome, and the data showed that the lysosome signals in the cytoplasm significantly increased in the AMPK-inhibited oocytes (Fig. 5E), and the general lysosome signal intensity was much higher than the control group (1 vs. 3.97 ± 1.1, *p* < 0.01) (Fig. 5F). These data suggested that AMPK activity was important for ER and Golgi distribution during oocyte maturation.

## Discussion

AMPK is one critical regulator for metabolism and is related with mitochondria, autophagy and other multiple cellular processes. In present study, we investigated the potential relationship between AMPK expression and aging in oocytes, and we explored the roles of AMPK in oocytes. Our data indicated that AMPK expressed altered in aged oocytes and AMPK inhibition induced increased ROS level, mitochondria dysfunction and the aberrant organelle function, which contributed the oocyte maturation defects with mouse model.

We first examined the expression of AMPK in mouse oocytes and the data showed that AMPK expressed at the different stage of oocyte meiotic maturation. The localization pattern was consistent with other cell models of previous studies [37]. Next, we explored the relationship between AMPK and oocyte aging, and interestingly AMPK expression decreased in the aging oocytes, which was similar with many other metabolic proteins. Since during oocyte aging, many proteins showed decreased level, which contribute to the declined cellular function and low oocyte developmental competence [11]. Especially, mitochondria distribution and functions were aberrant in aged oocytes [39]. The low AMPK expression level might be related with the decreased metabolism in the oocytes. To further testify this, we treated the oocytes with AMPK inhibitor COM C, and the results showed that inhibition of AMPK further aggravated the declined oocyte quality caused by aging, due to the fragmentation from morphology, which further confirm that potential relationship between AMPK and oocyte aging.

We then inhibited AMPK and the results showed that loss of AMPK activity caused the failure of oocyte polar body extrusion, indicating the essential roles of AMPK for oocyte maturation. To explore how AMPK functions in mouse oocytes, we examined the ROS level, and the results indicated that loss of AMPK activity induced increased ROS level, which is the typical index for the occurrence of oxidative stress. Previous studies also showed that AMPK could reduce oxidative stress in many models (Barone, Di Domenico, Perluigi, & Butterfield [2]),. In oocytes, it is shown that during postovulatory oocyte aging, the AMPK expression is related with oxidative stress under the kaempferol treatment [28, 38]. PKD1 is reported to regulate AMPK in many models [35], for example, it is shown that PKD1 could inhibit the phosphorylation of AMPK at Ser(485/491) in skeletal muscle cells [7]. Besides, Insulin and PMA treatment increased p-AMPK level through PKD1 in HepG2 cells [1]. Therefore, to confirm our finding, we also inhibited PKD1 and similar results were observed, which further support our finding. Indeed, during oocyte aging, ROS-mediated oxidative stress is one important cause for the declined oocyte quality, and our results connected the relation between AMPK with oxidative stress during aging. Mitochondria is most close organelle with ROS as introduced above, and our data showed that inhibition of AMPK caused aberrant mitochondria distribution, low ATP production and even decreased mitochondria number due to mtDNA data, which may be the main reason for AMPK on oxidative stress control. Numerus studies provided multiple evidence for the relationship between AMPK and mitochondria from different models [10]. In oocytes, it is shown that α1AMPK depletion caused aberrant mitochondria physiology and junctional protein expression in mouse oocytes [3]. As the energy sensor, activation of AMPK by AICAR increased mitochondria function and promotes bovine oocyte developmental competence (Takeo, Abe, Shirasuna, Kuwayama, & Iwata [26]). Therefore, AMPK may regulate mitochondria function to prevent oxidative stress during mouse oocyte maturation.

Besides its roles on mitochondria-related metabolism management, our results also exhibited that the AMPK inhibition caused a large proportion of organelles such as ER and Golgi apparatus showing with the aberrant distribution. Organelle dysfunction could lead to oocyte maturation defects. For example, exposure to mycotoxin citrinin or zearalenone both disrupted Golgi apparatus distribution and function, which induced the failure of mouse oocyte maturation (M. H. Sun et al [24, 25]; Y. Wang, Xing, Chen, & Sun [31]). Previous studies showed that AMPK could moderate the phosphorylation of GBF1 for mitotic Golgi disassembly [18]. Metformin, as the activator of AMPK, could promote anti-tumor immunity though its effects on ER-related PD-L1 [5]. Moreover, it is shown that the localization of ER could be affected by mitochondria; similarly, previous studies also indicated that mitochondria functions also could altered the Golgi apparatus fragmentation (Wenzel, Elfmark, Stenmark, & Raiborg [33]). The alternation in mitochondrial function was intimately connected to abnormal ER and Golgi function, and enhancing perturbation in the normal ER or Golgi function might lead to apoptotic cell death mediated by mitochondria (Khatoon, Pahuja, & Parvez [14]). Therefore, the disruption of ER and Golgi in AMPK-inhibited oocytes may be due to the effects of AMPK on mitochondria. Moreover, we also observed the increased lysosome after AMPK inhibition. Lysosome is the place for autophagy, while autophagy response to the oxidative stress. This further confirmed the roles of AMPK on oxidative stress control. A recent study showed that AMPK could phosphorylate FNIP1 for lysosomal and mitochondrial biogenesis [17]. Moreover, it is shown that lysosome acts as a critical hub for AMPK and mTORC1 [4]. Besides, several evidences indicate that AMPK signaling pathway could coordinates autophagy and metabolism [20], and its roes on autophagy was through the phosphorylation mTROC1, ULK1 and others [16]. Our finding was consistent with these findings and showed the conserved roles of AMPK on lysosome-related autophagy.

## Conclusions

In summary, our data showed that AMPK expressed in mouse oocytes and was related with oocyte aging, and AMPK may affect mitochondria function for oxidative stress and maintain organelle ER/Golgi function in mouse oocytes.

## Data Availability

No datasets were generated or analysed during the current study.

## References

[1] Allen KM, Coughlan KA, Mahmood FN, Valentine RJ, Ruderman NB, Saha AK (2017). The effects of troglitazone on AMPK in HepG2 cells. Arch Biochem Biophys.

[2] Barone E, Di Domenico F, Perluigi M, Butterfield DA (2021). The interplay among oxidative stress, brain insulin resistance and AMPK dysfunction contribute to neurodegeneration in type 2 diabetes and Alzheimer disease. Free Radic Biol Med.

[3] Bertoldo MJ, Guibert E, Faure M, Rame C, Foretz M, Viollet B, Froment P (2015). Specific deletion of AMP-activated protein kinase (alpha1AMPK) in murine oocytes alters junctional protein expression and mitochondrial physiology. PLoS ONE.

[4] Carroll B, Dunlop EA (2017). The lysosome: a crucial hub for AMPK and mTORC1 signalling. Biochem J.

[5] Cha JH, Yang WH, Xia W, Wei Y, Chan LC, Lim SO, Hung MC (2018). Metformin promotes Antitumor Immunity via Endoplasmic-Reticulum-Associated degradation of PD-L1. Mol Cell.

[6] Chauhan S, Singh AP, Rana AC, Kumar S, Kumar R, Singh J, Kumar D (2023). Natural activators of AMPK signaling: potential role in the management of type-2 diabetes. J Diabetes Metab Disord.

[7] Coughlan KA, Valentine RJ, Sudit BS, Allen K, Dagon Y, Kahn BB, Saha AK (2016). PKD1 inhibits AMPKalpha2 through phosphorylation of serine 491 and impairs insulin signaling in skeletal muscle cells. J Biol Chem.

[8] Duan X, Sun SC (2019). Actin cytoskeleton dynamics in mammalian oocyte meiosis. Biol Reprod.

[9] Feng Y, Chen Y, Wu X, Chen J, Zhou Q, Liu B, Yi C. Interplay of energy metabolism and autophagy. Autophagy. 2023;1–11. 10.1080/15548627.2023.2247300. 10.1080/15548627.2023.2247300 PMC10761056 37594406

[10] Herzig S, Shaw RJ (2018). AMPK: guardian of metabolism and mitochondrial homeostasis. Nat Rev Mol Cell Biol.

[11] Huang J, Chen P, Jia L, Li T, Yang X, Liang Q, Zhou C (2023). Multi-omics Analysis reveals translational landscapes and regulations in mouse and human oocyte aging. Adv Sci (Weinh).

[12] Ju JQ, Pan ZN, Zhang KH, Ji YM, Liu JC, Sun SC (2023). Mcrs1 regulates G2/M transition and spindle assembly during mouse oocyte meiosis. EMBO Rep.

[13] Kang X, Wang J, Yan L (2023). Endoplasmic reticulum in oocytes: spatiotemporal distribution and function. J Assist Reprod Genet.

[14] Khatoon R, Pahuja M, Parvez S (2020). Cross Talk between Mitochondria and other targets in Alzheimer’s Disease. J Environ Pathol Toxicol Oncol.

[15] Lan M, Zhang Y, Wan X, Pan MH, Xu Y, Sun SC (2020). Melatonin ameliorates ochratoxin A-induced oxidative stress and apoptosis in porcine oocytes. Environ Pollut.

[16] Li Y, Chen Y (2019). AMPK and Autophagy. Adv Exp Med Biol.

[17] Malik N, Ferreira BI, Hollstein PE, Curtis SD, Trefts E, Novak W, Shaw S (2023). Induction of lysosomal and mitochondrial biogenesis by AMPK phosphorylation of FNIP1. Science.

[18] Mao L, Li N, Guo Y, Xu X, Gao L, Xu Y, Liu W (2013). AMPK phosphorylates GBF1 for mitotic golgi disassembly. J Cell Sci.

[19] Metur SP, Klionsky DJ (2023). Nutrient-dependent signaling pathways that control autophagy in yeast. FEBS Lett.

[20] Mihaylova MM, Shaw RJ (2011). The AMPK signalling pathway coordinates cell growth, autophagy and metabolism. Nat Cell Biol.

[21] Pan ZN, Pan MH, Sun MH, Li XH, Zhang Y, Sun SC (2020). RAB7 GTPase regulates actin dynamics for DRP1-mediated mitochondria function and spindle migration in mouse oocyte meiosis. FASEB J.

[22] Shan MM, Zou YJ, Pan ZN, Zhang HL, Xu Y, Ju JQ, Sun SC. Kinesin motor KIFC1 is required for tubulin acetylation and actin-dependent spindle migration in mouse oocyte meiosis. Development. 2022;149(5). 10.1242/dev.200231. 10.1242/dev.200231 35142352

[23] Silva BR, Silva JRV (2023). Mechanisms of action of non-enzymatic antioxidants to control oxidative stress during in vitro follicle growth, oocyte maturation, and embryo development. Anim Reprod Sci.

[24] Sun GY, Gong S, Kong QQ, Li ZB, Wang J, Xu MT, Tan JH (2020). Role of AMP-activated protein kinase during postovulatory aging of mouse oocytesdagger. Biol Reprod.

[25] Sun MH, Li XH, Xu Y, Xu Y, Pan ZN, Sun SC. Citrinin exposure disrupts organelle distribution and functions in mouse oocytes. Environ Res. 2020;185:109476. 10.1016/j.envres.2020.109476. 10.1016/j.envres.2020.109476 32278162

[26] Takeo S, Abe T, Shirasuna K, Kuwayama T, Iwata H (2015). Effect of 5-aminoimidazole-4-carboxamide ribonucleoside on the mitochondrial function and developmental ability of bovine oocytes. Theriogenology.

[27] Tian Y, Liu X, Pei X, Gao H, Pan P, Yang Y. Mechanism of mitochondrial Homeostasis Controlling Ovarian Physiology. Endocrinology. 2022;164(1). 10.1210/endocr/bqac189. 10.1210/endocr/bqac189 36378567

[28] Wang L, Tang J, Wang L, Tan F, Song H, Zhou J, Li F (2021). Oxidative stress in oocyte aging and female reproduction. J Cell Physiol.

[29] Wang X, Tan X, Zhang J, Wu J, Shi H (2023). The emerging roles of MAPK-AMPK in ferroptosis regulatory network. Cell Commun Signal.

[30] Wang Y, Pan ZN, Xing CH, Zhang HL, Sun SC (2022). Nivalenol affects spindle formation and organelle functions during mouse oocyte maturation. Toxicol Appl Pharmacol.

[31] Wang Y, Xing CH, Chen S, Sun SC (2021). Zearalenone exposure impairs organelle function during porcine oocyte meiotic maturation. Theriogenology.

[32] Wang Y, Xing CH, Zhang HL, Pan ZN, Sun SC (2021). Exposure to nivalenol declines mouse oocyte quality via inducing oxidative stress-related apoptosis and DNA damage. Biol Reprod.

[33] Wenzel EM, Elfmark LA, Stenmark H, Raiborg C. ER as master regulator of membrane trafficking and organelle function. J Cell Biol. 2022;221(10). 10.1083/jcb.202205135. 10.1083/jcb.202205135 PMC9481738 36108241

[34] Wu SL, Ju JQ, Ji YM, Zhang HL, Zou YJ, Sun SC (2023). Exposure to acrylamide induces zygotic genome activation defects of mouse embryos. Food Chem Toxicol.

[35] Xiang H, Zhang J, Lin C, Zhang L, Liu B, Ouyang L (2020). Targeting autophagy-related protein kinases for potential therapeutic purpose. Acta Pharm Sin B.

[36] Xu Y, Sun MH, Xu Y, Ju JQ, Pan MH, Pan ZN, Sun SC (2020). Nonylphenol exposure affects mouse oocyte quality by inducing spindle defects and mitochondria dysfunction. Environ Pollut.

[37] Ya R, Downs SM (2014). Perturbing microtubule integrity blocks AMP-activated protein kinase-induced meiotic resumption in cultured mouse oocytes. Zygote.

[38] Zeng ZC, Jiang J, Wang XJ, Wei KN, Liang HS, Zeng LX, Wang HL (2022). Kaempferol ameliorates in-vitro and in-vivo postovulatory oocyte ageing in mice. Reprod Biomed Online.

[39] Zhang C, Dong X, Yuan X, Song J, Wang J, Yin X, Wu K (2023). Post-ovulatory aging affects mitochondria, spindle and protein metabolism in mouse oocytes. Reproduction.

[40] Zou YJ, Shan MM, Wan X, Liu JC, Zhang KH, Ju JQ, Sun SC (2022). Kinesin KIF15 regulates tubulin acetylation and spindle assembly checkpoint in mouse oocyte meiosis. Cell Mol Life Sci.

